# Lived experiences of spiritual emotional intelligence: a *Bhagavad Gitā*-based indigenous model of psychological wellbeing

**DOI:** 10.3389/fpsyg.2025.1730103

**Published:** 2025-12-11

**Authors:** Sai Kiran Gannamraju, Venkatesh H. Chembrolu

**Affiliations:** Indian Knowledge System and Mental Health Applications Centre (IKSMHA), Indian Institute of Technology, Mandi, Himachal Pradesh, India

**Keywords:** spiritual emotional intelligence (SEI), *Bhagavad Gitā*, Indian psychology, emotional intelligence, positive psychology, psychological capital, wellbeing

## Abstract

This study explores the lived experiences of Spiritual Emotional Intelligence (SEI), a psychological framework rooted in the *Bhagavad Gitā*, a foundational text of Indian spiritual philosophy. Building on a previously proposed model comprising five dimensions—ā*tma-bodha* (self-awareness), *samatva* (equanimity), *niṣkāma-karma* (non-attached action), *Dharma* (virtuous duty), and *bhakti* (devotion)—we conducted a qualitative study with 26 participants from diverse cultural and professional backgrounds. Semi-structured interviews examined how individuals apply these principles in daily life. Thematic analysis revealed enhanced self-reflection, emotional regulation, intrinsic motivation, ethical decision-making, and a deepened sense of meaning. Even neutral participants resonated with *Gitā*-based values such as mindfulness, non-attachment, and integrity, demonstrating their universal psychological applicability beyond religious identity. By integrating spiritual and emotional competencies, SEI fosters psychological flourishing and moral purpose. Grounding emotional intelligence in an indigenous Indian worldview, this research expands current understandings of positive psychology and culturally responsive wellbeing. It contributes to indigenous psychology by demonstrating how the *Bhagavad Gitā*, as a traditional knowledge system, offers a culturally grounded intervention for psychological flourishing in diverse communities, bridging Eastern and global contexts.

## Introduction

Psychology has increasingly turned toward the study of human flourishing, defined as optimal wellbeing encompassing both pleasurable experiences and meaningful engagement. Within this shift, Positive Psychology has emphasized constructs such as Emotional Intelligence (EI) and Psychological Capital (PsyCap) as foundational to flourishing. EI, popularized by Goleman, involves recognizing and managing one's own and others' emotions, fostering healthy relationships, and regulating stress. PsyCap comprises four positive psychological resources: Hope, self-efficacy, Resilience, and Optimism (HERO), which enhance adaptive functioning and mental health.

However, mainstream models of wellbeing, primarily rooted in Western secular contexts, have been critiqued for sidelining spiritual and existential concerns. Spiritual intelligence has emerged as a complementary construct, highlighting the capacity to find deeper meaning, act virtuously, and engage with life from a transcendent perspective. Research shows that religious and spiritual engagement often correlates with better mental health, as it offers comfort, purpose, and hope during times of stress. As Koenig (2009) observed, spirituality often fosters resilience by focusing on meaning rather than pathology.

In parallel, psychology has begun to integrate non-Western knowledge systems. The Indian tradition offers deep psychological insights, notably through the *Bhagavad Gitā*, a 700-verse dialogue dated around the second century BCE. The *Gitā* addresses themes of duty, identity, virtue, and emotion through a counseling exchange between Lord Kṛṣṇa and Arjuna. Scholars have noted the *Gitā*'s relevance as a psychological guide, emphasizing discipline, self-mastery, and equanimity. Teachings on sense control, non-attached action, higher devotion, and balance amid dualities echo modern constructs such as mindfulness, intrinsic motivation, cognitive appraisal, and resilience.

This study responds to the call in *Frontiers in Psychology's* Research Topic on *Psychological Wellbeing in Indigenous Communities: Traditional Knowledge and Cultural Interventions* (Research Topic ID: 71741), which invites scholarship exploring how traditional knowledge systems can serve as culturally grounded resources for psychological flourishing. While rooted in the *Bhagavad Gitā*, a cornerstone of Indian spiritual philosophy, this study also aligns with broader global efforts to recognize and integrate indigenous frameworks. For example, Aboriginal, Native American, and African traditions emphasize the interconnection between spirituality, resilience, and community wellbeing.

Despite this, Indian psychology has often been overshadowed by Western frameworks imported from abroad. The Indian Psychology movement and calls to decolonize psychology (Rao and Paranjpe, 2016) advocate for deeper engagement with indigenous systems to enhance global models of mental health. Importantly, the *Gitā* can be seen as part of India's traditional knowledge system, paralleling how other indigenous communities use sacred texts, oral narratives, and ritual practices to guide emotional resilience and community wellbeing. In this way, the present study contributes to the broader dialogue on how indigenous spiritual frameworks can inform culturally grounded models of psychological flourishing.

Spiritual Emotional Intelligence (SEI) emerges at the confluence of emotional and spiritual development. In earlier work, we proposed a conceptual model of SEI grounded in the *Gitā* and linked to EI and PsyCap. The model comprises five dimensions: ā*tma-bodha* (self-awareness of the true Self), *samatva* (equanimity), *niṣkāma-karma* (non-attached action), *Dharma* (virtuous duty), and *bhakti* (devotion or spiritual love). These correspond with EI competencies, such as regulation, empathy, and motivation, and may also enhance PsyCap traits; for instance, *samatva* and *bhakti* could reinforce hope and resilience.

However, despite theoretical promise, empirical research on how these *Gitā*-based principles manifest in daily life is sparse. Most prior studies have been conceptual or quantitative. Gayathri and Meenakshi (2012) found parallels between *Gitā* teachings and models of emotional regulation, including impulse control and empathy. Singh and Raina (2015) developed an anāsakti (non-attachment) scale, which is linked to wellbeing. Pandya (2023) implemented a *Gitā*-based counseling program for older diaspora adults, observing a reduction in stress and an improvement in quality of life. Dabas and Singh (2018) reported that *Gitā* training improved optimism and reduced stress in Indian students. While these findings are promising, little is known about how individuals apply principles like equanimity, devotion, or non-attached action in everyday contexts. Lived-experience perspectives are crucial to refining culturally grounded emotional intelligence frameworks.

This study addresses that gap by investigating the lived experiences of SEI through qualitative interviews. We engaged 26 participants from diverse backgrounds to explore how *Gitā*-based principles are applied and the outcomes that result. Using thematic analysis of personal narratives, we examined how individuals enact ā*tma-bodha, samatva, niṣkāma-karma, Dharma*, and *bhakti*, and how these relate to emotional wellbeing, relationships, and personal growth. By moving from conceptual proposal to experiential validation, this research bridges Indic spiritual psychology with contemporary health science. It situates SEI as part of the broader movement to incorporate indigenous wisdom traditions into the science of human flourishing.

## Literature review

### *Bhagavad Gitā* and psychological wellbeing

The *Bhagavad Gitā* has long been recognized as a text with profound psychological insights, addressing Arjuna's anxiety and grief and offering strategies to overcome despair through self-knowledge, duty, and devotion. Bhatia et al. (2013) note parallels with modern psychotherapy, including detachment from negative thoughts, meditation, and finding purpose in duty. Teachings on sense mastery resonate with cognitive-behavioral and mindfulness approaches, while the *sāttvic* personality (calm, self-controlled, pure) aligns with emotional stability in positive psychology. Empirical studies of the *triguṇa* show *sāttva* predicts higher wellbeing, whereas *rajas* and *tamas* correlate with distress. Indian psychology has sought to bridge such concepts with empirical tools: Wolf (1999) developed the Vedic Personality Inventory, Singh and Raina (2015) validated an *anāsakti* (non-attachment) test linking it with emotional regulation, and Bhawuk (2011) argued for culturally sensitive approaches to spirituality, noting that qualitative methods reveal how people apply principles like equanimity in daily life. More recently, Indian psychology has been situated within broader decolonial conversations, with Dhillon (2023) highlighting how integrative frameworks grounded in the *Bhagavad Gitā* are being revisited to align traditional Indic wisdom with modern psychological paradigms. This underscores the urgency of empirically articulating frameworks like SEI that honor Indic philosophical sources while engaging contemporary psychology.

### Emotional intelligence, spiritual intelligence, and PsyCap

Emotional Intelligence (EI), popularized by Goleman (1996), encompasses the ability to perceive, understand, and regulate emotions (Mayer et al., 2004), and supports effective leadership, mental health, and healthy relationships. Eastern contemplative traditions have long emphasized emotional mastery, with mindfulness now a mainstream method for enhancing self-awareness and impulse control. Spiritual Intelligence (SI) extends this to meaning-making, ego transcendence, and virtue (Emmons, 2000). King and DeCicco (2009) proposed critical existential thinking and conscious state expansion, while Amram and Dryer (2008) created the ISIS scale to assess mindfulness, faith, and interconnectedness.

Empirical research confirms SI's adaptive value: Jankowski et al. (2022) found it central to flourishing; Ajele et al. (2021) linked SI and mindfulness with less depression and greater wellbeing; and Singla et al. (2021) demonstrated that teachers with higher SI reported stronger PsyCap and work-life quality. Pinto et al. (2024), in a recent global scoping review, described SI as a “gateway to mental health and resilience,” emphasizing its preventive potential in education and healthcare. Similarly, Korkut and Çetin (2025) found SI positively predicted professional values among nursing students in Türkiye, demonstrating both the validity and applied significance of SI measures in contemporary contexts. These findings suggest synergy between EI, SI, and PsyCap (hope, efficacy, resilience, optimism; Luthans et al., 2007), where spiritual frameworks reinforce psychological resources.

### Prior *Gitā*-based models and empirical studies

Several *Gitā*-based approaches to mental health exist. Sharma and Batra (2019) emphasized detachment and devotion for stress reduction; Kathuria and Awasthy (2022) linked *Gitā* and Biblical teachings to integrity and empathy; Kanwal (2020) applied “spiritual intelligence training” for psychosocial rehabilitation; and Pandya (2023) demonstrated reduced stress in elders through *Gitā*-based counseling. Despite these advances, most studies examine specific interventions or remain theoretical (Bhawuk, 2011). Raina et al. compared *Gitā* readers and non-readers, finding differences in values and maturity; yet, detailed accounts of everyday application, such as practicing non-attachment in exams or cultivating equanimity at work, remain scarce.

Collecting such narratives can refine the SEI framework, highlight challenges, and strengthen the case for integrating *Gitā*-inspired practices into positive psychology paradigms. Notably, the Global Flourishing Study (VanderWeele et al., 2025), which surveyed over 200,000 participants across 22 countries, demonstrates the contemporary centrality of spirituality and religion in flourishing outcomes. This large-scale evidence lends weight to the present study, which explores qualitatively how *Gitā*-rooted SEI principles manifest in the lived experiences of diverse individuals.

Despite the expanding evidence for Emotional and Spiritual Intelligence in relation to flourishing, and the recognition of Indian psychology's relevance to wellbeing, key gaps remain. Most existing SI and positive psychology frameworks emphasize mindfulness, meaning making, and resilience, but they rarely incorporate devotion (*bhakti*), surrender, or scripturally grounded transcendence as psychological resources. Similarly, while the *Bhagavad Gitā* is acknowledged as a spiritual text with therapeutic relevance, few empirical studies capture how its principles are lived and practiced in contemporary contexts. This study addresses these gaps by examining Spiritual Emotional Intelligence (SEI), a model derived from the *Bhagavad Gitā* that integrates five dimensions (ā*tma-bodha, samatva, niṣkāma-karma, Dharma*, and *bhakti*). Through qualitative interviews, the research highlights how *bhakti* and Indic philosophical insights uniquely enrich emotional intelligence and positive psychology, offering a culturally grounded, holistic pathway to psychological flourishing.

### Theoretical framework: *Gitā*-based SEI model

The SEI model, derived from the *Bhagavad Gitā*, identifies five dimensions: ā*tma-bodha* (self-awareness), *samatva* (equanimity), *niṣkāma-karma* (detached action), *Dharma* (virtue), and *bhakti* (devotion; Prabhupada, 1972). These integrate spiritual principles with emotional and psychological competencies and link directly with Emotional Intelligence and Psychological Capital (see Table 1).

**Table 1:** Dimensions of SEI and their psychological linkages.

**SEI dimension**	***Bhagavad Gitā* basis**	**Psychological construct**	**Contribution to EI/PsyCap**
*$\bar{\text{A}}$tma-bodha* (self-awareness of the soul)	*Gitā* 2.20—ā*tman* is eternal and unchanging	Mindfulness, meta-cognition	EI: self-awareness & regulation; PsyCap: resilience, efficacy
*Samatva* (equanimity)	*Gitā* 2.14, 2.48—treat pleasure and pain alike	Emotional balance, acceptance	EI: self-regulation; PsyCap: resilience, optimism
*Niṣkāma-karma* (action without attachment to results)	*Gitā* 2.47—duty without attachment to fruits	Intrinsic motivation, acceptance-commitment	EI: motivation, regulation; PsyCap: hope, efficacy, optimism
*Dharma* (virtue and responsibility)	*Gitā* 3.35—acting in line with role and universal ethics	Values-based living, character strengths	EI: empathy, social skills; PsyCap: resilience, moral efficacy
*Bhakti* (devotion and spiritual anchoring)	*Gitā* 18.58—refuge in the divine	Spiritual coping, gratitude, self-transcendence	EI: empathy, transcendence; PsyCap: hope, optimism, resilience

Together, these five dimensions form a holistic model of SEI rooted in the *Bhagavad Gitā*. They are not isolated traits but interdependent: self-awareness (ā*tma-bodha*) supports detachment in action (*niṣkāma-karma*), while devotion (*bhakti*) reinforces equanimity (*samatva*) through trust in a higher purpose. This synthesis reflects what Positive Psychology terms flourishing: living with inner balance, meaning, and practical engagement. The model thus provides a culturally grounded framework that bridges Indic spirituality with contemporary constructs of emotional intelligence and psychological capital.

### Research questions

Drawing from the literature and SEI framework, this study explores three questions:

1. **Integration of** ***Git*ā** **Principles**: How do individuals apply SEI teachings (e.g., equanimity for stress, *bhakti* in loss) in daily life domains such as relationships, stress management, and growth?
2. **Lived Experiences of SEI Dimensions**: How are ā*tma-bodha, samatva, niṣkāma-karma, Dharma*, and *bhakti* manifested across diverse groups (e.g., students vs. homemakers, neutral vs. religious)?
3. **Perceived Impacts on Wellbeing**: Do SEI practices enhance resilience, flourishing, stress reduction, and relational harmony, aligning with constructs like Psychological Capital?

## Method

### Research design

This study employed a qualitative design, guided by phenomenological and thematic analysis, to explore the lived experiences of Spiritual Emotional Intelligence (SEI). Semi-structured interviews allowed participants to share personal stories in their own words. Ethical approval was obtained from an institutional review board, and informed consent (verbal and written) was secured from all participants prior to data collection.

### Participants

The sample consisted of 26 participants (8 male, 18 female), aged 20–59 years (mean age ≈ 36). They represented diverse professions and life contexts, including students, homemakers, lawyers, dieticians, software engineers, corporate leaders, and academics. Approximately two-thirds were based in India (e.g., Mumbai, Hyderabad, Vadodara, Visakhapatnam), while the remaining one-third were from the Indian diaspora or international contexts. These included a German kindergarten teacher living in Munich (P03) and a Ukrainian PhD scholar in psychology (P14), as well as participants in London, the USA, and Australia. This geographical spread enriched the study with both Indian and cross-cultural perspectives. Full demographic details are provided in Appendix A.

Participants also varied in spiritual orientation:

- **Devout practitioners/“spiritual-and-religious”** (≈18) affiliated with *bhakti*-yoga traditions, reporting daily mantra meditation, scriptural study, and vegetarian practices.
- **Moderate/“spiritual-but-not-religious”** participants (≈5) engaged in yoga, mindfulness, or occasional chanting, resonating with *Gitā* values without strict rituals.
- **Neutral participants** (3) had little or no religious practice, emphasizing ethical living and karma (action) over devotion.

Given the predominance of Indian participants, future studies should examine SEI in other indigenous communities to enhance cross-cultural generalizability. The sample's diversity in spiritual orientation, from devout practitioners to secular participants, demonstrates the adaptability of SEI principles across varying cultural and religio-spiritual profiles.

**Sampling and inclusion criteria**. Participants were recruited using a purposive sampling approach through professional and spiritual networks, community referrals, and snowballing to ensure diversity in gender, age, occupation, and spiritual orientation. Inclusion criteria required that participants were 18 years or older, fluent in English, and willing to engage in a 25–35minute recorded interview.

**Data adequacy and saturation**. Recruitment continued until thematic saturation was reached—no new subthemes appeared across three consecutive interviews after the 23rd interview. This decision followed an information-power rationale (Malterud et al., 2016), ensuring the final sample size of 26 captured sufficient conceptual depth and variation across spiritual orientations.

### Data collection

Interviews ranged from 30 to 40 min and were conducted entirely in English via Zoom. The interview guide explored participants' backgrounds, emotional challenges, understanding of SEI dimensions (ā*tma-bodha, samatva, niṣkāma-karma, Dharma*, and *bhakti*), the role of spirituality or values, and perceived outcomes, including resilience, flourishing, and relational harmony. Neutral participants were asked about their values rather than their religious practices, while devout practitioners often spontaneously cited *Gitā* verses or shared devotional experiences. All interviews were audio-recorded with permission, transcribed verbatim, and anonymized.

The semi-structured interview guide included open-ended prompts exploring emotional challenges, spirituality, and the five SEI dimensions. As participants varied in their spiritual orientation (devout, moderate, secular), some prompts were adapted for contextual relevance. The complete list of sample questions is presented in Appendix B.

### Data analysis

We analyzed the data using thematic analysis, following Braun and Clarke's (2006) guidelines. Given the predefined SEI dimensions, our approach combined deductive (theory-driven) and inductive (data-driven) coding.

**Coding Process:** The first cycle of coding was structured around the five SEI dimensions as parent nodes. Using Microsoft Excel, we created columns for Participant ID, Parent Node, Subtheme, Quote, and Memo. Segments were coded under relevant dimensions (e.g., observing anger without reacting → ā*tma-bodha*; staying calm under criticism → *samatva*). Content outside the five categories (e.g., outcomes, reflections) was coded separately for contextual analysis. Although specialized qualitative software (e.g., MAXQDA, NVivo, Atlas.ti) can assist in large-scale projects, given the modest sample size (26 transcripts) and the structured nature of the SEI coding framework, Microsoft Excel was sufficient for systematic coding, organization, and theme development. This approach, using Microsoft Excel, is well-suited for small to medium-sized qualitative datasets where transparency, manual immersion, and structured coding are prioritized over automation. This approach has precedent in qualitative research where transparency and manual immersion in the data are prioritized.

**Subtheme Development:** Within each parent node, inductive analysis generated subthemes. For example, under ā*tma-bodha*, participants described “pausing before reacting,” labeled as *reflection before reaction*. Under the concept of *Dharma*, accounts of “*staying honest under pressure*” formed the subtheme of integrity under pressure. Subthemes were refined iteratively, yielding 15–20 distinct subthemes per dimension.

**Memos and Reflexivity:** Throughout coding, analytic memos captured insights, theme relationships, and quotes. For instance, one memo noted: “Daily meditation (*bhakti*) reinforces equanimity (*samatva*).” Another observed: “Neutral participants resonate with karma-yoga but hesitate about *bhakti*.” The interdisciplinary team (psychology and Hindu philosophy) discussed coding differences, enhancing reflexivity, and reliability.

**Trustworthiness:** Two researchers independently coded five transcripts, discussed discrepancies to reach consensus, and refined a shared codebook used for the remaining data. We maintained an audit trail comprising iterative versions of the codebook, memos, and analytic decisions and engaged in continuous analytic memoing. Member-checking was undertaken *in situ* during interviews through reflective summaries (e.g., “Have I captured this correctly?”). Patterns were examined across participants to support triangulation of accounts and strengthen interpretive credibility.

To ensure methodological transparency, we also reflected on how our positionality influenced the analysis. Both authors are of Indian descent with training in psychology and Eastern philosophy, bringing cultural familiarity with the *Bhagavad Gitā* alongside academic grounding in Positive Psychology (e.g., Seligman, 2011). The first author's lived engagement with the *Gitā* informed nuanced interpretations of constructs such as *Dharma* and *titikṣā* (e.g., BG 2.14, 2.47). In contrast, the second author's background in Indian psychology added contextual accuracy.

We recognized the risk of bias, including over-identification with indigenous constructs or the unconscious imposition of Western frameworks. To mitigate this, we used investigator triangulation through cross-coding and dialogue, maintained reflexive journaling to track interpretive shifts, and cross-validated readings of *Gitā* concepts (e.g., *samatva, titikṣā*) with empirical applications (e.g., Dabas and Singh, 2018; Pandya, 2023). In line with broader reflexivity standards (Berger, 2015), this process strengthened epistemic reflexivity and enhanced the credibility of our culturally grounded synthesis.

**Reflexivity:** The authors' familiarity with Indian philosophical psychology sensitized the analysis to metaphors and practices based on the *Bhagavad Gitā*. To minimize potential confirmation bias, dual-coding and discrepant-case discussions were employed, and all interpretive decisions were logged through analytic memos. Reflexive journaling documented shifts where participant narratives refined rather than confirmed the a priori SEI dimensions. This process ensured that the framework evolved from lived data rather than theoretical preconceptions, aligning with standards for reflexive thematic analysis (Braun and Clarke, 2021; Berger, 2015).

**Thematic Synthesis:** Subthemes were synthesized by reviewing the frequency, intensity, and uniqueness of the data. For example, overlaps between ā*tma-bodha* and *samatva* showed that self-awareness often preceded equanimity. Triangulation compared participant accounts with one another and with literature. Informal member checks were conducted during interviews (“It sounds like you are saying X, is that right?”). Two researchers independently coded a subset of transcripts, achieving high agreement; discrepancies were resolved through discussion and refinement of the codebook. All analyses used anonymized data, and quotes are presented with participant IDs or descriptors only.

### Ethical considerations

Given the spiritual nature of the topic, interviews carried potential for sensitive disclosures (e.g., religious struggles, personal values). We approached all interactions with cultural sensitivity and respect. Participants were clearly informed that they could skip questions or withdraw at any time. The consent form and verbal explanation clearly specified the voluntary nature of participation, outlined how data would be used for research and potential publication, and offered the option to receive a summary of results as a form of debriefing and reciprocity.

To protect confidentiality, all names and identifiable details were replaced with pseudonyms or generic descriptors in transcripts and reports. During interviews, particular care was taken when emotional topics emerged (e.g., coping with bereavement through spirituality). The interviewer paused to ensure participants were comfortable, creating space for them to share only as much as they wished.

This study was reviewed and approved by the Institutional Ethics Committee of the Indian Institute of Technology Mandi (Approval No.: IITM/IEC(H)/2023/VD/P2). Participants provided verbal informed consent, which was audio-recorded prior to participation in accordance with the approved protocol. No major ethical issues arose, and many participants reported that reflecting on the spiritual and emotional aspects of their lives was a meaningful experience; several described the interview as both positive and therapeutic. Ethical safeguards, including voluntary participation, confidentiality, informed consent, institutional approval, and sensitivity in handling disclosures, were strictly followed, ensuring the integrity of the study and the wellbeing of participants.

## Results

Findings are presented across the five dimensions of the *Bhagavad Gitā*–based Spiritual Emotional Intelligence (SEI) model: ā*tma-bodha* (self-awareness), *samatva* (equanimity), *niṣkāma-karma* (detached action), *Dharma* (virtue/duty), and *bhakti* (devotion/anchoring). Each dimension is explored through participants' lived experiences, illustrated with direct quotes, and interpreted in light of existing psychological frameworks such as Emotional Intelligence, Positive Psychology, and Psychological Capital (PsyCap). To highlight key patterns, tables summarize exemplar quotes and outcomes. Variations across age, gender, culture, and spirituality are also noted, followed by a discussion of overlaps between dimensions.

### *Ātma-bodha* (self-awareness of the soul)

Participants emphasized the importance of inner observation, emotional awareness, and self-checking, which they experienced as crucial for wellbeing, consistent with *Bhagavad Gitā* 2.20, which highlights the distinction between the eternal self and the mind and body. For many, this awareness often appeared to emerge through pausing and reflecting before action. A software leader (P06 AVP) shared, “*Previously it was more of a reactive nature… My tone softened, my approach started becoming more responsive rather than reactive*,” reflecting mindful regulation and resilience as described in Psychological Capital (PsyCap). A homemaker (P17) stated, “*I just controlled myself for one day… by the next day I felt it was not necessary, because it was her perception. It does not change who my father is*,” showing emotional detachment and stability. Students described managing anxiety through grounding: “*Before, when there were ups and downs, I used to react according to the situations… But after having that understanding through spirituality… I have started to be calm with every situation, whether it be a good situation or a bad situation, you know, just offer it all to Radha Gopinath at the end of the day*.” (P04, Student/Entrepreneur/Athlete), resonating with cognitive-behavioral strategies while rooted in spiritual identity. A coach and devotee (P10) described a body-based practice for self-awareness and calming emotions: “*I call it feel, breathe, and comfort. So, I feel what is happening in my body… I name the feeling, then I breathe and calm that energy down*.” A psychologist (P14) highlighted the role of naming and validating emotions as part of self-awareness: “*I always try to acknowledge my emotions and name them… to realize that, okay, I am feeling this and this is like anger or this is frustration. And after I have done this, I also validate them. I say that, yeah, okay, this emotion is fine to feel right now*.” In cross-cultural contexts, ā*tma-bodha* promoted emotional balance and stability of identity. A German kindergarten teacher (P03) reflected, “*I could see that when you are in the mode of goodness… you can act with a solution, with calm. We were all in passion, but my husband kept so calm*.” (P03, German teacher), highlighting resilience amid cultural demands.

Across profiles, ā*tma-bodha* was frequently described as foundational, and was associated with self-regulation, stress reduction, and a grounded self-concept that strengthens the efficacy and resilience components of psychological capital (PsyCap).

### *Samatva* (equanimity)

Equanimity: remaining steady in success and failure (*Gitā* 2.48), was frequently mentioned as a coping mechanism. Many described *samatva* as emotional balance in the face of dualities. A finance professional (P19, Fund Manager) emphasized: “*Whether you have a great day or a bad day, can you have the same mental framework and remain equipoised?*” For homemakers, it meant cultivating steadiness rather than reacting. A nutritionist (P23) shared, “*I do not feel so much stress now after having spent a few years in Krishna consciousness*.” Students echoed this; P16 admitted, “*I mean, I feel angry at the start. But then I am like, I cannot do anything. So, it is okay*.”

Even secular participants framed equanimity as valuable. A lawyer (P21) said, “*I never react. And even if I react, it's always very softly and calmly… Even if someone has to show anger to me, my anger comes out in tears*.” This illustrates *samatva's* universality beyond devotional contexts. In sum, *samatva* was described as a buffer against volatility, and appeared to support resilience and optimism.

### *Niṣkāma-karma* (detached action)

*Niṣkāma-karma*: acting without attachment to results (*Gitā* 2.47), emerged strongly in work, family, and service contexts. In professional life, participants noted reduced stress when focusing on effort rather than outcome. An entrepreneur (P26) reflected, “*Even if I am worried about the result, when I get into the process, that worry goes away… I really enjoy the process*.” A postgraduate student pursuing her PhD in the United States (P22) shared, “*Now, when I go through something, I immediately understand, okay, this is what is happening with me, and then I try to deal with it*.” Such detachment *was described as supporting* perseverance and emotional regulation. In family roles, detachment meant serving without expectation. A homemaker (P07) said, “*Do everything but with detachment. Putting Krishna in between every relationship and serving as if you are serving Krishna and not the person in front of you*.” Another participant (P08, Monk–Physicist) noted, “*What used to bother me 100% seventeen years back… certainly 50–60, maybe 70% has gone down. It evolves like a spiral; sometimes up and down but overall moving upward*.” A secular participant (P16, Student, Mumbai), reflected a similar ethic of detached action without devotional framing: “*I've learned that if you just finish your work on time and not delay it, you feel light in the long run… procrastination only makes it worse*.”

Volunteering also reflected *niṣkāma-karma*. A daily practitioner (P01, Team Leader) explained, “*In temple service, I do not think of recognition, just service*.” Across various contexts, participants described *niṣkāma-karma* as nurturing, and prior work has reported associations wi*th* intrinsic motivation, reducing stress, promoting humility, and cultivating hope, all qualities that are linked to Psychological Capital (PsyCap).

### *Dharma* (virtue and responsibility)

*Dharma*, or fulfilling one's duty with integrity, was often described as a guiding compass. A professor (P05) stressed service integrity: “*See, sometimes I should not say when our devotees come and want to do some program, I go and I find the toilet unclean, I clean those toilets also. I do not mind*.” This reflects integrity under pressure, linked to PsyCap's efficacy and optimism in ethical action. Family duty was equally highlighted. A homemaker (P17) explained, “*If he [her husband] is very unwell, then I say [no to other commitments]. But if it is his normal unwell, then he has to deal with it… But then, I do not let it affect me. I know it is my duty now*.” Such responsibility was experienced as bringing meaning and resilience. Similarly, secular participants interpreted *Dharma* through humanistic values rather than religious duty. “*Honestly, I am not very religious. For me, spirituality is a thousand percent karma—just doing good and keeping everyone happy*.” (P13, Housewife, Secular).

Young professionals emphasized purposeful work. A lawyer (P21) shared how she gave up a prestigious opportunity in London to return and support her father's business: “*I just closed my eyes and pictured my grandfather, who always taught us that family is above everything. And then if you take that decision, everything else flows*.” This illustrates, in her account, a dharmic sense of duty and a perceived link to eudaimonic wellbeing. Diaspora participants described *Dharma* as cultural continuity within daily life. A German teacher (P03) explained this through practices of sharing prasādam (sanctified food) and devotional singing: “*I give them Prasadam, and sometimes when I sing God's names, babies do hear*.”

### *Bhakti* (devotion and spiritual anchoring)

*Bhakti*: devotion to the divine, was central for the daily practices of most practitioners. Many described how surrender and devotional focus were reported to reduce anxiety. A senior engineer (P25) reflected, “*When I see with a spiritual perspective… something we think is a big problem becomes negligible… that will give mental peace*.” Students also relied on *bhakti* for strength. A postgraduate living in London (P09) said, “…*when you do chanting, you call Krishna. Krishna hears you. So, obviously, when we are stressed or when I bit down… chanting and reading helped me out*.” Such devotional anchoring enhanced efficacy and reduced anxiety.

*Bhakti* further offered community support and meaning-making. A homemaker (P17) shared, “*There are a lot of associations of devotees… it gives us a lot of hope, that whatever is being taught is being practiced, and it helps a lot*.” Even “spiritual but not religious” participants resonated with *bhakti*. An entrepreneur (P26) explained, “*I chant daily one round, not much. And I pray in the morning, I go to the temple, I offer flowers. And you know, just those five minutes of starting my day… I am starting my day with the faith that [Krishna] is just going to send the right answer. And it will come through me and maybe not from me*.” Such trust was associated with optimism, illustrating in these accounts how *bhakti* can serve as a secure existential anchor that reinforces hope, resilience, and relational harmony.

### Variations across participants

Differences across age, gender, and culture shaped SEI expressions. Younger participants emphasized coping with academic stress (e.g., detachment from exam results, P04), while older participants highlighted family and caregiving duties (e.g., P17 caring for her ill husband). Women often framed SEI in relational contexts, whereas men stressed professional or service domains. Cultural contrasts also emerged: Indian participants associated SEI with daily temple and family practices, while diaspora participants emphasized the continuity of their identity, as seen in P3's teaching of Indian values in Germany. A Ukrainian participant (P14) underscored self-awareness through reflection, showing SEI's adaptability beyond Indian culture.

Spiritual orientation further shaped expression. Devout *bhakti*-yoga practitioners focused on *bhakti* and *Dharma*, while secular participants leaned on karma and equanimity. P21 (lawyer, spiritual but not religious) said, “*I never react. And even if I react, it's always very softly and calmly… Even if someone has to show anger to me, my anger comes out in tears*.” This embodied equanimity without ritual devotion. P02 (designer, secular) emphasized karma as ethical work. Overall, SEI's flexibility was evident in these narratives: while devotion anchored practitioners, secular individuals accessed its psychological benefits through universal principles such as equanimity and self-awareness.

### Interconnections of SEI pillars

Thematic analysis revealed substantial overlaps between SEI dimensions. $\bar{\text{A}}$*tma-bodha* (self-awareness) often enabled *samatva* (equanimity), as participants described being aware of anger and responding with calmness. *Bhakti* similarly reinforced *samatva*, with regular chanting said to increase inner balance. *Niṣkāma-karma* and *Dharma* frequently intertwined: acting without attachment was closely aligned with fulfilling duty, as professionals described ethical work (*Dharma*) carried out without concern for recognition (*niṣkāma-karma*). Participants also highlighted feedback loops, *bhakti* deepened *Dharma* (“service as devotion”), while *samatva* stabilized *niṣkāma-karma* by reducing anxiety about outcomes. These interconnections suggest that SEI may operate less as discrete traits and more as a holistic ecosystem, where cultivating one dimension reinforces others and fosters flourishing.

Across participants, the SEI framework illuminated how *Bhagavad Gitā* principles are actively lived. $\bar{\text{A}}$*tma-bodha* fostered mindful self-awareness, *samatva* balanced emotions, *niṣkāma-karma* reduced anxiety about results, *Dharma* grounded ethical purpose, and *bhakti* anchored resilience through devotion. Together, these dimensions were described as aligning with **PsyCap** facets of hope, efficacy, resilience, and optimism. Variations across age, gender, culture, and spirituality revealed the universality and adaptability of SEI, while the interconnections between pillars underscored its holistic character. These findings extend both Positive Psychology and Indian Psychology, offering a culturally grounded model of emotional and spiritual flourishing. The key themes and outcomes from the study are summarized in Table 2.

**Table 2:** Summary table of themes and outcomes.

**SEI pillar**	**Subtheme**	**Exemplar quote**	**Psychological outcome**
*$\bar{\text{A}}$tma-bodha* (self-awareness)	Pause before reaction	“*Previously, it was more of a reactive nature. My tone softened, my approach started becoming more responsive rather than reactive*.” (P06 AVP Software)	Emotional regulation, resilience
*$\bar{\text{A}}$tma-bodha*	Witnessing the mind	*I just controlled myself for 1 day… by the next day, I felt it was not necessary, because it was her perception. It does not change who my father is*.” (P17, Homemaker)	Identity stability, reduced distress
*$\bar{\text{A}}$tma-bodha*	Anxiety recognition	“*Before, when there were ups and downs, I used to react according to the situations… However, after having that understanding through spirituality… I have started to be calm with every situation, whether it be a good situation or a bad situation*.” (P04 Student)	Stress reduction, coping skills
*Samatva* (equanimity)	Market resilience	“*Whether you have a great day or a bad day, can you have the same mental framework and remain equipoised?*” (P19 Fund Manager)	Resilience, realistic optimism
*Samatva*	Emotional steadiness in family life	“*I do not feel so much stress now after having spent a few years in Krishna consciousness*.” (P23 nutritionist)	Relational harmony; reduced reactivity
*Samatva*	acceptance/letting go	“*I mean, I feel angry at the start. But then I am like I cannot do anything. So, it is okay*.” (P16 Student)	Growth mindset
*Niṣkāma-karma* (detached action)	Work detachment	“*Even if I am worried about the result, when I get into the process, that worry goes away for me… the worry about the result is more external, like what will other people think of me. But the work that I am doing is what I love doing*.” (P26 Entrepreneur)	Reduced anxiety, intrinsic motivation
*Niṣkāma-karma*	Family service	“*Do everything but with detachment. Putting Krishna in between every relationship and serving as if you are serving Krishna and not the person in front of you*” (P07 Homemaker)	Humility, relational harmony
*Dharma* (Virtue/Duty)	Honesty	“P05 (Professor) *emphasized serving with integrity, even doing humble tasks like cleaning toilets himself when needed*.”	Integrity, efficacy
*Dharma*	Family duty	“*If he [her husband] is very unwell, then I say [no to other commitments]. But if it is his normal unwell, then he has to deal with it… But then, I do not let it affect me. I know it is my duty now*.” (P17 Homemaker)	Resilience, meaning
*Dharma*	Professional service	“*I came back to handle [the family business] and support him. And now I am doing a mixture of law and business as well*.” (P21 Lawyer)	Purpose-driven action; value alignment; eudaimonic wellbeing
*Bhakti* (Devotion)	Surrender coping	“*When I see with a spiritual perspective… something we think is a big problem becomes negligible… that gives mental peace*.” (P25 Engineer)	Stress relief, clarity, satisfaction
*Bhakti*	Devotional preparation	“*When I feel lonely or when I feel a bit down, I do chanting. And afterwards I feel a bit better*.” (P09 Student)	Efficacy, reduced anxiety
*Bhakti*	Community support	“*There are a lot of associations of devotees… it gives us a lot of hope, that whatever is being taught is being practiced, and it helps a lot*.” (P17 Homemaker)	Social resilience
*Bhakti*	Higher trust	“*I trust a higher force guiding me*.” (P26 Entrepreneur)	Optimism, meaning

## Integration with contemporary wellbeing frameworks (EI, PsyCap, positive psychology)

Themes of self-awareness, equanimity, non-attachment, virtue, and devotion align with the principles of positive psychology and emotional intelligence. Here, the findings are interpreted through the frameworks of Psychological Capital (PsyCap), Emotional Intelligence (EI), and flourishing.

### SEI and psychological capital (HERO resources)

Psychological Capital (PsyCap) is defined by the HERO resources—Hope, Efficacy, Resilience, and Optimism (Luthans et al., 2007). Participants' experiences showed that SEI naturally reinforced these resources.

**Hope:** *Bhakti* and surrender provided future-oriented hope. P19 (Fund Manager) described how chanting and *Gitā* reflections help him stay emotionally stable amid market uncertainties, reminding himself to tolerate ups and downs and remain equipoised. Such spiritual framing preserved hope by preventing despair in adverse conditions, aligning with prior work that suggests spiritual worldviews enhance hope and purpose in the face of adversity (Koenig, 2012).

**Efficacy:** Detachment and *Dharma* strengthened self-efficacy. Through ā*tma-bodha*, participants developed realistic self-views, enhancing clarity and success. Acting ethically built moral efficacy: “*I try to see what best others can do, how can I make them happy rather than me keeping in the center, not satisfying my ego*” (P05, Professor). Faith further reduced self-doubt, allowing bold action without ego.

**Resilience:** *Samatva* and *bhakti* emerged as key tools for resilience. Participants calmly handled criticism and accepted dual outcomes (P01, Team Leader). Devotion and community provided coping resources, from chanting to alleviate anxiety (P09, Student in London) to accepting difficulties as part of Krishna's will and karma (P17, Homemaker). A finance professional (P18) reframed stress through a probability mindset: “*Before getting into the outcome, have I given my 100%? If yes, then I try not to look at the outcome*.” Across participants, SEI dimensions created a “resilience toolkit”: self-awareness flagged negative states, *Dharma* motivated perseverance, detachment buffered disappointment, and devotion infused meaning.

**Optimism:** SEI cultivated what Frankl termed “tragic optimism”—confidence in growth through suffering rather than naive positivity. By focusing on duty and surrender, participants trusted that outcomes would unfold meaningfully. *Bhakti* reinforced optimism through the perception of divine care, fostering a secure yet realistic outlook.

Together, these findings suggest SEI amplifies PsyCap's HERO resources through cognitive, emotional, and spiritual pathways, complementing quantitative studies (e.g., Singla et al., 2021) with rich narrative evidence.

### SEI and emotional intelligence (EI) competencies

Emotional Intelligence (EI), as defined by Mayer and Salovey and popularized by Goleman, encompasses self-awareness, self-regulation, motivation, empathy, and social skills. Participant experiences demonstrated that SEI reinforced and extended each of these domains. $\bar{\text{A}}$*tma-bodha* mapped to EI's self-awareness, *samatva* to self-regulation, *niṣkāma-karma* and *Dharma* to motivation, and *bhakti*/*Dharma* to empathy and social skills.

**Self-Awareness:** Participants displayed emotional maturity and value-based discernment. A student (P24) shared, “*Others are too much concerned with face, cuteness, and Instagrams… I am lucky I am into Krishna consciousness*,” reflecting a grounded identity shaped by spiritual values. Practices such as chanting and scriptural reflection supported emotional differentiation.

**Self-Regulation:** *Samatva* helped participants stay composed under provocation: “*I do not see any problem as a big problem. These things seem to be very normal. I mean, I do not… that is there*” (P05, Professor). *Niṣkāma-karma* reduced fear and greed, stabilizing emotions, resonating with mindfulness, and reframing practices in EI training but grounded in *Gitā* principles.

**Motivation:** Duty and service fostered intrinsic motivation. P20 (Designer) explained, “*First of all, I think about the people before the project*,” prioritizing responsibility over recognition. Similarly, P22 (PhD scholar) reflected, “*One thing that I am always driven toward is serving… if I do not see if it is helping to make somebody's life better, I do not feel fulfilled and content*.” This shows motivation enriched with altruism and devotion.

**Empathy & Social Skills:** *Dharma* and *Bhakti* Shaped Relational Ethics. P25 (Principal Engineer) reflected, “*I do not want to be unnecessarily ambitious… I just want to be balanced*,” expressing humility and fairness. *Bhakti* further nurtured compassion, patience, and listening, echoing Amram and Dryer's (2008) identification of compassion and humility as spiritual intelligence traits overlapping with EI.

Crucially, SEI addressed EI's critique of moral neutrality. Whereas, EI skills can be used manipulatively, SEI integrates *Dharma* and *bhakti*, embedding competencies in ethical and altruistic purpose. As P14 (Psychologist, Ukraine) explained, “*Psychology is more focused on individuality… You need to put yourself first, think about yourself. But in Krishna consciousness, you learn to be altruistic and see others, see Krishna's needs. It puts you on a more altruistic platform and allows you to be part of a bigger thing*.”

### Alignment with wellbeing and flourishing frameworks

Seligman's PERMA model defines flourishing through *Positive Emotion, Engagement, Relationships, Meaning, and Accomplishment*. Participants' experiences demonstrated strong alignment with these dimensions.

**Positive Emotion:** “*When I feel lonely or when I feel bit down, I do chanting. And afterwards I feel bit better. Because… when you do chanting you call Krishna. Krishna hears you*” (P09, Student in London). Burns et al. (2021) demonstrate that flourishing protects mental health; our findings illustrate spiritual practices as a preventive measure for maintaining mental health.

**Engagement:** Removing ego distractions fostered flow-like states, with participants describing a loss of track of time in service or chanting classic PERMA engagement.

**Relationships:** *Dharma* and *bhakti* improved family and community bonds, fostering forgiveness and empathy, in line with PERMA's focus on positive relationships.

**Meaning:** Nearly all participants found purpose through duty, service, or devotion. P18 (Finance Professional) shared, “*The only aim I have is to try to make people happy… maybe monetarily, maybe emotionally, maybe by giving time*.” Such *bhakti*-oriented altruism grounds meaning beyond career outcomes, resonating with Frankl (1985) and Park (2010).

**Accomplishment:** Though not primary, SEI practices indirectly improved performance, health, and discipline, supporting goal attainment.

Overall, SEI fostered both hedonic (happiness and calmness) and eudaimonic (virtue and meaning) flourishing, consistent with VanderWeele's (2017) holistic model. Indian psychology provides further evidence: non-attachment correlates with wellbeing (Singh and Raina, 2015), and sattva is associated with mental health (Khanna et al., 2013). A unique SEI contribution is the spiritual connection itself—*bhakti* evoked awe, love, and peace, dimensions often missing in secular models. This aligns with empirical findings on spirituality's contribution to flourishing (Jankowski et al., 2022) and reinforces broader calls to integrate spirituality into public health frameworks (Oman and Syme, 2018). The major gaps in existing wellbeing frameworks and the corresponding contributions of the SEI model are presented in Table 3.

**Table 3:** Gaps in wellbeing models and SEI contributions.

**Model/framework**	**Gap**	**SEI contribution**	**Evidence (participant quote)**
Emotional intelligence (EI)	Morally neutral; limited attention to meaning	*Dharma* and *bhakti* embed EI skills in ethical and altruistic frameworks	P05 (Professor) emphasized integrity, even doing humble tasks like cleaning toilets. P22 (PhD Scholar) shared, “*Whenever I feel restless or anything, I start chanting… it calms me down, and it gives me the space to clearly think*.”
Psychological capital (PsyCap)	HERO resources are secular and individualistic	*Bhakti* gives transcendent hope; *niṣkāma-karma* builds resilience; ā*tma-bodha* enhances efficacy	“*When playing sports… we gave our absolute best, but still the results were not in our favor. Surrendering to Krishna then made a great deal of sense because… There must be some cosmic reason to this that we did not win*.” (P04, Student/Entrepreneur/Athlete)
PERMA flourishing	Limited structured spirituality; lacks transcendence	SEI adds *bhakti* (spiritual anchoring), *niṣkāma-karma* (detachment), *samatva* (equanimity)	P20 (Designer) reflected, “*If this project is not coming, then no issues… let us just focus on the opportunities which I am going to get if I try even more, even better*.” P17 (Homemaker) described caring for her unwell husband as her duty, supported by chanting and performing Mangal Aarti. P18 (Finance Professional) emphasized meaning as “*making people happy*” through help and service
Positive psychology (general)	Criticized as Western-centric, hedonic, and individualistic	SEI integrates Indic virtues (non-attachment, humility, forbearance)	P01 (Team Leader) described the transformation from arrogance to humility
Wellbeing interventions	Often rely on mindfulness/CBT; limited cultural resonance	SEI offers *Gitā*-rooted, culturally meaningful practices	“*When I feel lonely or when I feel bit down, I do chanting. And afterwards I feel bit better. Because… when you do chanting you call Krishna. Krishna hears you*.” (P09, Student in London)

## Discussion

### Summary of key findings

This study explored how Spiritual Emotional Intelligence (SEI), grounded in the *Bhagavad Gitā*, is lived and practiced. Analysis of 26 interviews identified five core dimensions: ā*tma-bodha* (self-awareness), *samatva* (equanimity), *niṣkāma-karma* (non-attached action), *Dharma* (duty/virtue), and *bhakti* (devotion). These dimensions aligned with established psychological models, including Psychological Capital's HERO resources (hope, efficacy, resilience, and optimism), Emotional Intelligence competencies (awareness, regulation, motivation, empathy, and social skills), and Positive Psychology's PERMA model (positive emotion, engagement, relationships, meaning, and accomplishment). Notably, SEI *appears* to extend these frameworks by integrating emotional skills into ethical and spiritual values, *offering* a culturally grounded model that addresses critiques of individualistic approaches and contributes to broader theories of flourishing. These findings align with the *Global Flourishing Study* (VanderWeele et al., 2025), which demonstrated the strong association between spirituality/religion and flourishing across 22 countries. Whereas, this global survey established that spirituality predicts flourishing, the present study illustrates, via participants' accounts, how individuals reported integrating, through practices such as self-awareness (ā*tma-bodha*), equanimity (*samatva*), and devotion (*bhakti*), individuals integrate Indic spiritual-emotional resources into stress management, relationships, and personal growth. To further clarify how SEI extends existing frameworks, it is helpful to briefly position it in relation to established models of Emotional and Spiritual Intelligence.

### SEI vis-à-vis SI and EI

While Emotional Intelligence (EI) focuses on the perception, understanding, and regulation of emotions, and Spiritual Intelligence (SI) emphasizes meaning-making, self-transcendence, and higher purpose (e.g., Emmons, 2000; Zohar et al., 2000), Spiritual Emotional Intelligence (SEI), as conceptualized in this study, is articulated here as a relational integration of these capacities. SEI comprises five *Bhagavad Gitā*–grounded competencies—ā*tma-bodha* (self-awareness), *samatva* (equanimity), *niṣkāma-karma* (non-attached action), *Dharma* (value-aligned duty), and *bhakti* (devotional/self-transcending love)—that together may inform how emotions are appraised, regulated, and ethically directed toward eudaimonic wellbeing. Thus, SEI is not a simple sum of SI and EI, but a culturally anchored synthesis in which emotional skills are ethically and existentially scaffolded by Indic philosophical principles, linking self-regulation with self-realization.

### Interpretive diversity

We acknowledge the diverse interpretations of *Dharma, samatva*, and *niṣkāma-karma* within Indian philosophical traditions. For example, *Advaita Vedānta* emphasizes non-dual insight, *Bhakti* traditions center on loving devotion, and *Karma-yoga* prioritizes duty performed without attachment. The present articulation of SEI follows a *Bhagavad Gitā*–centric synthesis while remaining compatible with these broader strands, situating SEI within India's interpretive diversity rather than a single doctrinal view.

### Theoretical implications and new insights

**Validation of the SEI Framework:** The findings supported the credibility of the SEI framework in this sample, showing how each dimension translated into observable attitudes and behaviors that enhanced participants' ability to manage emotional challenges. $\bar{\text{A}}$*tma-bodha* was expressed through mindful self-observation and a reduction in impulsivity. As one entrepreneur noted, “*Yesterday I had a meeting… she was coming across so aggressively saying it is my show. But I was so impressed with myself that I did not react*” (P11, Entrepreneur), illustrating how spiritual self-awareness fosters wise responses. *Samatva* was described as reflecting balance amid success and failure, consistent with the *Gitā*'s teaching and Weber's (2020) notion of equanimity in mental wellbeing. *Niṣkāma-karma* was associated with resilience and intrinsic motivation, echoing Singh and Raina's (2015) findings on the benefits of non-attachment. *Dharma* grounds meaning and virtue in daily life, aligning with Peterson and Seligman's (2004) virtue-based wellbeing. *Bhakti* was reported as providing a sense of secure attachment and hope, consistent with Koenig's research on spirituality and coping (Koenig, 2012). Together, these accounts lend support to the SEI model's theoretical plausibility in this context and show how *Gitā*-based principles enrich psychological constructs of flourishing. Recent SI studies reinforce this view: Pinto et al. (2024) mapped SI as a gateway to resilience and mental health, while (Korkut and Çetin 2025) found SI positively predicted professional values among nursing students. However, these frameworks often lack explicit integration of devotion and *Dharma*. The current findings suggest SEI contributes precisely to this dimension, adding culturally rooted practices that deepen SI's explanatory and applied potential.

**Model refinement:** While many narratives converged with the five SEI dimensions, several accounts refined and extended them. For instance, secular participants enacted *bhakti*-like other-regard through community service rather than theistic devotion, and *samatva* was sometimes cultivated via non-religious mindfulness or reflective journaling. These nuances expand SEI's theoretical scope, showing that its core competencies can manifest across devotional and secular orientations. Such refinements inform future measurement and intervention design by underscoring SEI's flexibility as a culturally anchored yet universally applicable construct.

**Integration of Indic Wisdom with Psychology:** Participants applied *Gitā* principles dynamically, for example, using *Gitā* 2.37 to cope with job loss, suggesting adaptability and affirming calls to integrate non-Western paradigms into psychology. Indigenous concepts such as *anāsakti* (detachment) and ś*raddhā* (faith) emerged as practical tools for resilience, supporting Bhatia and Priya's (2018) critique of neglected constructs and Rao and Paranjpe's (2016) call for culturally grounded psychology. As one PhD scholar in Australia shared, “*Sometimes I feel my PhD itself is Krishna's plan. I still remember the day I got my offer letter, it was Janmashtami. I had tears in my eyes; it felt like a call from a higher power*” (P15, PhD Scholar in Australia). This illustrates, in this participant's account, how faith and surrender may function *as* psychological resources. These findings resonate with the broader decolonial turn in Indian psychology (Dhillon, 2023), which advocates moving beyond Western-derived models toward frameworks grounded in indigenous epistemologies. SEI contributes to this movement by illustrating, in these data, how *Bhagavad Gitā*-based concepts such as *niṣkāma-karma* and *bhakti* function as lived psychological resources, addressing calls for culturally rooted approaches to mental health and flourishing.

**Holistic Character of SEI:** SEI appeared holistic in participants' accounts, with multiple dimensions often engaged simultaneously: witnessing anger (ā*tma-bodha*) enabled calm response (*samatva*), reinforced by prayer (*bhakti*) and principled restraint (*Dharma*). This interweaving *supports viewing* SEI as a yoga-like system of integrated faculties. For example, a corporate leader highlighted how inner stability supports family wellbeing: “*I think both family harmony and inner peace, but I think inner peace is first for me. Because if I am okay internally, then I will be able to be part of and contribute to family harmony*.” (P12, Director). An emergent theme was compassion (karuṇā), frequently arising from the interaction of self-awareness, equanimity, and devotion, suggesting it may deserve explicit inclusion in future refinements.

**Culturally Specific vs. Universal:** Self-awareness, equanimity, and altruistic duty emerged as near-universal ideals, valued even by neutral participants. *Bhakti* practices (e.g., chanting) were more culturally specific; yet their functions, community, hope, and surrender appear universal, adaptable to other faiths or secular devotion to higher purposes. For example, one housewife emphasized a neutral orientation, stating, “*Honestly, I am not very religious. And for me, spirituality is a thousand percent karma… if you tell me to go to the temple and sit and chant for two hours, I cannot do it. My karma has to be good, that is all I get*.” (P13, Neutral Housewife). This supports transpersonal psychology's perennial view while underscoring SEI's flexibility for multicultural applications.

**Religious and Secular Applicability:** Although many participants practiced theistic *bhakti*, parallel processes—such as purpose-based commitment, compassion practice, and values-guided service—also operated among secular and non-theistic participants. Thus, SEI's core competencies—*samatva* (equanimity), *niṣkāma-karma* (non-attachment to outcomes), *Dharma* (value-aligned action), ā*tma-bodha* (reflexive awareness), and *bhakti* (other-regard)—can be expressed through non-religious frames, including humanistic ethics, mindfulness, and community service. This *suggests that* SEI is not confined to faith contexts but represents a broader human capacity for ethical-emotional integration and purposeful living, thereby extending its relevance to both religious and secular applications.

The *Bhagavad Gitā*-based SEI model addresses these limitations across both religious and secular contexts. Evidence from the current study demonstrating how SEI fills gaps in existing emotional and spiritual intelligence models is provided in Table 4.

**Table 4:** How SEI fills gaps in existing emotional and spiritual intelligence models.

**Existing gap/limitation**	**Evidence/support from literature**	**How SEI model fills/addresses it**
Lack of integration between spirituality and emotional intelligence.	Spirituality is often treated separately from emotional regulation in SI models; weak integration noted (Jisna, 2024).	SEI integrates spiritual and emotional processes through dimensions such as ā*tma-bodha* and *samatva*, demonstrating how spirituality directly supports emotional growth.
Models are abstract/lack cultural grounding.	Many SI frameworks are decontextualized and abstract (King, 2008; Hyde, 2004); calls for culturally specific models have been made (Moberg, 2002; MacDonald, 2000).	SEI grounds itself in the principles of the *Bhagavad Gitā* (e.g., *Dharma, niṣkāma-karma, bhakti*), ensuring cultural specificity and lived applicability.
Limited measurement tools/low psychometric validity.	Existing SI scales show cultural bias or psychometric weaknesses (King and DeCicco, 2009; Amram and Dryer, 2008).	SEI offers a framework for developing psychometrically valid, culturally embedded measures aligned with *Gitā*-based indicators.
Cultural and demographic bias.	Most SI studies are Western-centric, which limits their generalizability (Macdonald and Friedman, 2002).	SEI is rooted in Indian philosophical foundations and designed for cross-cultural adaptability and empirical testing.
Focus on coping/crisis, not flourishing.	SI research often emphasizes coping with illness/suffering (Koenig, 2018; Pargament et al., 2013).	SEI emphasizes proactive spiritual cultivation for flourishing, resilience, and growth, consistent with flourishing-oriented models (Seligman, 2011; VanderWeele, 2017).
Lack of developmental or longitudinal focus.	Few models explore how SI competencies evolve over time (MacDonald, 2000).	SEI enables developmental analysis, for example, growth in ā*tma-bodha* or deepening of *samatva* through sādhana across the life span.
Insufficient triangulation in research.	Many studies rely only on self-report, risking social desirability bias (Moberg, 2002).	SEI invites mixed-method validation, combining behavioral, physiological, and peer-report evidence with qualitative insights.
Lack of structured interventions.	SI interventions often lack theoretical consistency and rigorous evaluation (Zohar et al., 2000).	SEI provides a foundation for structured interventions based on its pillars, for example, *samatva* training or *bhakti*-based emotional awareness modules.

### Practical and clinical implications

The lived experiences of SEI have implications for mental health, education, organizational wellbeing, and the development of culturally congruent interventions.

**Counseling and Psychotherapy:** *Gitā*-based principles can support clients open to spiritual dialogue, especially those from Indian or Hindu backgrounds. Metaphors such as equanimity, duty, and non-attachment helped participants reframe stress. Teaching *niṣkāma-karma* (“perform effort sincerely and release the rest”) was described as reducing anxiety, while *samatva* stabilized reactivity. The *Gitā* itself proved therapeutic, as Arjuna's despair and Krishna's counsel mirror cognitive reframing (Gairola and Mishra, 2022). Counselors might ask, “What is your *Dharma* here?” or “How can you act without attachment?” One participant (P15, PhD scholar Australia) shared: “*When someone was yelling at me at work, instead of yelling back, I just asked, ‘Can we discuss this?' My previous version would have reacted, but now I try to understand why the person is behaving in a certain way*.” Others emphasized small practices: “*All these emotional things are like accidents… we have to take care of them just like a physical wound. Once I address that emotional accident, then I can remind myself of Krishna consciousness*.” (P10, Coach & Devotee). Neutral participants also benefited: “*I was diagnosed with acute depression… after therapy, I have changed a lot. I no longer wish to be there. Today I am much more comfortable, much more happier… as long as I am happy and as long as I am not bothering you intentionally, I am at peace*.” (P13, Housewife). This aligns with Pinto et al.'s (2024) call to use spiritual intelligence as a preventative strategy in healthcare and education. Embedding SEI-informed metaphors, such as equanimity and devotion, into counseling not only addresses immediate crises but also cultivates long-term resilience and flourishing.

**Wellbeing and Life Coaching:** SEI can enrich coaching, education, and workplace wellbeing. *Karma-yoga* (work as service) and *samatva* (balance) may reduce burnout, while leadership programs might draw on selfless leadership (Sharma and Batra, 2019). A manager confirmed that fairness improved trust. One corporate leader reflected: “*In the long run… if I have got a little bit of joy or happiness or peace, being associated with bhakti, with Krishna, I want to be that instrument for the people connected to me*.” (P12, Director). Schools can apply SEI in character education, exercises on success and failure build equanimity. Dabas and Singh (2018) found *Gitā* teachings increased optimism, echoed by a student: “*After surrendering to Krishna… it has become easier. Like, whatever happens… first I was a slave to my emotions. But now I have somewhat control over them*.” (P04, Student/Entrepreneur/Athlete).

**Spiritual Care and Holistic Health:** Many participants found chanting and prayer effective for stress relief. *Gitā*-based meditation or group recitation may aid stress management, as shown by Das et al. (2024). Participants reported an enhanced sense of meaning and outlook, echoing Frankl. “*If I am chanting attentively… after a good round of chanting, you always have some kind of realization that lifts you up further. It gives you spiritual strength*.” (P14, Psychologist, Ukraine). Patient preference remains key; neutral participants preferred non-religious framings.

**Community and Family Interventions:** Findings can inform community workshops (e.g., “*Gitā and Emotional Balance*”) linking scripture to psychological skills. Family counseling could integrate dharmic duties with communication strategies. One housewife emphasized: “*Every time it does not have to be the same boat, same page. It's okay to be individual. With my in-laws I prefer not to intervene, and with my parents I am very open… so, let's just keep it that way, live and let live*.” (P13, Housewife).

**Guidelines for Practitioners:** Practitioners should adapt terminology, e.g., “trusting life's process” instead of “surrender to God.” Narrative methods, such as Arjuna's dilemma, can foster insight. Small practices, such as breath meditation, gratitude, or reflective reading, were reported as effective by participants and are recommended; prior studies also suggest benefits for sustained regulation.

To align with the Research Topic's emphasis on practical applications, SEI can be structured into interventions such as *Gitā*-based counseling modules, youth wellbeing workshops utilizing *samatva* and karma yoga, or resilience programs in indigenous communities that emphasize service and mindfulness. These interventions can be evaluated through outcomes such as stress reduction, increased resilience, and enhanced meaning, using both qualitative narratives and standardized wellbeing measures.

### Comparison with existing literature

Our findings extend prior work on spirituality and emotional wellbeing. They support Lamba et al. (2023), who emphasized emotional intelligence from a *Gitā* perspective, and align with Dhillon (2023) on integrating ancient and contemporary psychology. The study adds depth to Singh and Raina (2015) by showing how non-attachment is practiced through letting go, and builds on Bhawuk (2011) by grounding *Gitā*-based psychology in lived narratives. It also strengthens positive psychology discourse, echoing VanderWeele (2017) and Jankowski et al. (2022) on spirituality's role in flourishing, while illustrating mechanisms, rituals, worldview, and social support through which spirituality reduces anxiety.

In contrast to secular EI training (Mayer et al., 2004; Goleman, 1995), this study suggests curricula enriched by spirituality may be more effective, echoing Walsh and Shapiro's (2006) call for meditative disciplines. *Bhakti* emerged in accounts *as* a distinctive addition, unlike broader SI theories (King and DeCicco, 2009; Amram and Dryer, 2008), highlighting personal devotion as a powerful emotional resource. Concerns that detachment undermines ambition are countered here, consistent with Kumar and Kumar (2013), who framed karma-yoga as enhancing positive work outcomes; participants demonstrated that detachment reduced anxiety while boosting performance. Overall, while many SI frameworks remain abstract or weakly integrated with emotional processes, the SEI model appears to offer a culturally grounded and distinctive contribution.

The SEI model is a theoretically grounded, culturally rooted yet adaptable framework that bridges spiritual and emotional competencies. Rooted in the *Bhagavad Gitā*, it retains universal applicability across cultures. It offers a foundation for validated scales and structured interventions, while drawing on indigenous wisdom to support the decolonization of psychology and expand global models of flourishing. Taken together, these insights position SEI as a bridge between classical Indic wisdom and contemporary wellbeing science, offering a replicable model for culturally grounded psychology.

### Cross-cultural and indigenous relevance

While this study focused on the *Bhagavad Gitā* as a foundation for SEI, its principles resonate with broader indigenous traditions worldwide. The emphasis on ā*tma-bodha* (self-awareness) and *samatva* (equanimity) parallels Native American storytelling and ritual practices that cultivate emotional resilience through the transmission of ancestral wisdom. Aboriginal Australian traditions of Dreamtime narratives similarly anchor identity and balance in relation to land and community, echoing *Dharma*'s call to live in harmony with cosmic order. African philosophies of Ubuntu, often encapsulated in the phrase “I am because we are,” reflect the relational aspects of *bhakti* and *Dharma*, emphasizing interconnectedness, service, and ethical responsibility. Even within tribal Indian communities, oral teachings and elder-guided rituals reflect principles of non-attached action and devotion as pathways to wellbeing. These parallels suggest that, although SEI is rooted in the Indic tradition, its core principles reflect globally resonant themes of spiritual and emotional integration. This makes SEI both culturally specific and broadly adaptable for diverse indigenous contexts seeking holistic mental health and flourishing. Thus, SEI not only contributes to indigenous Indian psychology but also opens a pathway for intercultural dialogue on spirituality and wellbeing, demonstrating that ancient wisdom can inform global psychological science.

## Limitations

This study provides valuable insights, but several limitations must be acknowledged.

The sample was predominantly English-speaking and devotional-leaning, which may limit transferability to non-devotional or vernacular populations. Interviews were mainly online and self-report-based. Future work should include vernacular interviews, a wider secular cohort, and non-urban contexts to examine how SEI manifests across settings.

Sample Characteristics. The sample (*N* = 26) was small and skewed toward spiritually inclined Hindu Indians, and thus was not statistically representative. Findings may be most relevant to populations culturally or spiritually aligned with the *Bhagavad Gitā*. Non-religious perspectives were limited, suggesting self-selection bias, those who volunteered likely already valued *Gitā* teachings.

### Social desirability and reporting bias

Given the interviewer's shared cultural background, some participants may have overemphasized the concept of success. Participants aware of the *Gitā*'s prestige may have overemphasized success in practicing equanimity or detachment. The interviewer's shared cultural background may also have encouraged alignment. While struggles were reported, a positive framing may still prevail. Triangulation with family or peer perspectives could have reduced this bias.

### Attribution challenges

Outcomes such as “less stress from chanting” cannot prove causality; other factors (e.g., career change, maturation) may contribute. Longitudinal designs are necessary to establish causal pathways and rule out confounding factors.

### Depth vs. breadth

Covering all five SEI dimensions offered a holistic picture but limited depth. Subthemes (e.g., forms of *Dharma*, filial, parental, professional) were collapsed for synthesis, which risked oversimplification.

### Reliance on self-report

Data were based on memory and subjective accounts, which may be fallible or selective. Still, consistency across participants lends credibility to patterns.

### Cultural embeddedness

Interpretations of terms such as ā*tma-bodha* or *Dharma* were framed within the *bhakti* and Vaiṣṇava commentarial traditions of the *Bhagavad Gitā*. Other philosophical schools (e.g., Advaita Vedānta, Karma Yoga traditions) may articulate these concepts differently; thus, the present findings represent one interpretive strand within the broader landscape of Indian thought.

### Observer bias

Researchers' positive regard for SEI may have amplified success stories while underemphasizing negatives. A few participants reported friction (e.g., family tension from “too much detachment”), underscoring risks of misapplication.

### Lack of long-term data

Outcomes were reported at the time of the interview, without the use of standardized pre- and post–measures. Future research should test SEI effects longitudinally using validated measures of wellbeing.

Finally, while the study provides a culturally rich account of SEI within a *Gitā*-based Hindu framework, this embeddedness also limits its scope. To enhance cross-cultural and indigenous relevance, future studies should examine how SEI principles align with or adapt to other indigenous frameworks (e.g., Native American, Aboriginal, or African traditions that emphasize community, ritual, or harmony with nature). This would clarify which dimensions (such as self-awareness and equanimity) are universal and which (such as *bhakti*) are culturally specific, thereby strengthening SEI's positioning as both an Indic contribution and a globally adaptable model of spiritual-emotional wellbeing.

## Future research directions

Several pathways for advancing research and application of Spiritual Emotional Intelligence (SEI) emerge from this study.

### Quantitative validation

A critical next step is the development of a psychometrically robust SEI scale. Items can be adapted from existing EI/SI measures while introducing unique domains such as *Dharma* and *bhakti* (e.g., “I remain calm whether praised or criticized”—*samatva*; “I strive to perform duties without expecting rewards”—*niṣkāma-karma*; “Prayer or gratitude helps me handle stress”—*bhakti*). Subthemes from this study provide candidate items. Validation should assess reliability, factorial structure, and convergent validity with flourishing, resilience, and mental health outcomes. Including *Dharma* and *bhakti* may strengthen predictive power, particularly in culturally relevant populations.

### Intervention studies

SEI-based interventions could be piloted in schools, workplaces, and community organizations. Modules may focus on mindfulness (ā*tma-bodha*), reframing dualities (*samatva*), service (*niṣkāma-karma*), values clarification (*Dharma*), and meditation or gratitude (*bhakti*). Evaluating outcomes such as stress reduction, resilience, performance, and relational harmony can clarify applied impact. Workplace applications may build on existing findings from karma yoga on organizational commitment. Adaptations for non-Hindu populations (e.g., reframing *bhakti* as gratitude, awe, or higher purpose) will enhance cross-cultural relevance.

### Longitudinal and developmental research

Cross-sectional narratives, while rich, cannot establish developmental change. Tracking SEI competencies across the lifespan may reveal how self-awareness, equanimity, or devotion mature over time, for instance, in neutral individuals who develop spiritual practices later in life. Studies with adolescents could examine how early exposure to *Dharma*, meditation, or equanimity shapes resilience and identity. Such work would complement prior calls for lifespan perspectives in Indian psychology (Misra and Gergen, 1993).

### Neuroscientific and behavioral correlates

Future research may combine qualitative insights with physiological and behavioral measures. For example, cortisol levels, heart-rate variability, or brain imaging could assess whether chanting or equanimity practices alter stress responses or activate self-regulation circuits. Prior meditation research (Desbordes et al., 2014) suggests such pathways; SEI provides culturally specific practices to extend these findings. Peer- or family-reported data could also triangulate self-report, reducing social desirability bias.

### Cross-cultural and indigenous relevance

While this study was rooted in Hindu/Indic participants, SEI may share parallels with indigenous and spiritual traditions worldwide. Narratives of surrender, community rituals, and faith-driven coping are familiar in Native American, Aboriginal, and African traditions. Comparative studies can explore universal and culture-specific expressions, positioning SEI as both locally grounded and globally adaptable. Such inquiry would strengthen cross-cultural psychology and support the decolonial turn in mental health research.

### Challenges and ethical nuances

Future studies must address the risks of misapplication, such as *samatva* being misunderstood as suppression, *Dharma* being stretched to justify overwork, or detachment being misread as indifference. The *Gitā* advocates moderation (e.g., balanced eating, sleeping, and working), and research should clarify how SEI can be taught without imbalance. Ethical guidelines for practitioners will be critical in preventing misuse.

### Policy and applied implications

SEI research can inform culturally sensitive interventions in mental health, education, and leadership. Policy frameworks for wellbeing may integrate indigenous wisdom traditions, such as incorporating *samatva* and *Dharma* into school curricula, or *bhakti* as gratitude-based spiritual care in health settings. Such applications align with ongoing efforts to decolonize psychology and embed traditional wisdom into formal wellbeing programs.

## Conclusion

This research highlights that culturally rooted spiritual frameworks can enhance emotional intelligence and overall wellbeing. The lived experiences of 26 participants demonstrate that the *Bhagavad Gitā*'s wisdom is not merely abstract poetry, but a practical guide to flourishing in today's world. By cultivating self-awareness of the higher self (ā*tma-bodha*), cultivating equanimity amid dualities (*samatva*), practicing selfless action (*niṣkāma-karma*), adhering to virtue (*Dharma*), and devoting oneself to the divine or higher ideal (*bhakti*), individuals develop resilience, meaning, and purpose.

Findings demonstrate that Spiritual Emotional Intelligence (SEI) is more than a theoretical construct; it is expressed in the effective management of workplace conflict, coping with loss, making ethical choices, and sustaining inner peace. SEI integrates spiritual values with psychological skills, creating a more balanced emotional intelligence that unites heart and soul. This model is particularly relevant in multicultural societies and for populations seeking meaning and transcendence in the context of mental health (Pargament et al., 2013).

Practically, SEI principles can guide therapists, educators, and leaders toward culturally sensitive, holistic interventions. For spiritual populations, framing equanimity, duty, or devotion in familiar terms enhances relatability, while neutral contexts can emphasize mindfulness, values, and compassion in non-religious language. Thus, SEI offers both flexibility and depth as a toolkit for wellbeing.

Importantly, this study highlights the value of lived experience in evaluating ancient wisdom. Participants' narratives reveal that “yoga of equanimity” and devotion are not antiquated but living resources for growth. In a world marked by stress and the search for meaning, the *Gitā*'s insights applied in modern lives emerge as catalysts for flourishing. By advancing such indigenous models, psychology can move toward inclusivity, weaving spirituality and science into a coherent narrative of health. Beyond these insights, the study also lays a foundation for future measurement and validation of SEI. Developing and testing a psychometrically robust SEI scale, including *Dharma* and *bhakti* as distinct domains, remains a critical next step. Longitudinal studies could further illuminate how SEI competencies evolve over time and across life stages, enabling a developmental psychology of spirituality that complements cross-sectional findings. Such empirical work would consolidate SEI as both a scholarly construct and a practical tool for interventions.

### Unique contributions of this study

Beyond affirming the theoretical promise of SEI, this study contributes three distinct advances. First, it documents lived experiences across neutral, spiritual-but-not-religious, and devout participants, grounding SEI in everyday life rather than relying solely on abstract theory or interventions. Second, it demonstrates how SEI expands mainstream models of wellbeing (EI, PsyCap, PERMA) by embedding ethical responsibility (*Dharma*) and devotional anchoring (*bhakti*), two dimensions often overlooked in secular psychology. Third, it highlights SEI's adaptability: while rooted in Indic wisdom, its universal themes of self-awareness, equanimity, and altruistic action resonate across cultural and non-religious contexts.

Taken together, these contributions position SEI as both a significant development within Indian psychology and a globally relevant framework for human flourishing, providing fertile ground for future measurement, interventions, and cross-cultural applications. In this way, SEI not only enriches Indian psychology but also extends the global flourishing paradigm of positive psychology by integrating ethical and spiritual anchors into the concept of emotional intelligence.

## Data Availability

The raw data supporting the conclusions of this article will be made available by the authors, without undue reservation.

## Appendix A

**Table A1:** Participant summary table.

**ID**	**Gender**	**Age**	**Profession/student**	**Spiritual orientation**	**Location**
P01	Male	45	Team Leader	Devout	Diaspora (Munich)
P02	Female	31	Brand Designer	Neutral	India (Mumbai)
P03	Female	55	Kindergarten Teacher	Devout	Diaspora (Munich)
P04	Female	20	Student (Bachelors, Finance BM 3rd Yr)	Devout	India (Mumbai)
P05	Male	42	Professor	Devout	India (Hyderabad)
P06	Male	46	AVP, Software Company	Devout	India (Bangalore)
P07	Female	54	Housewife	Devout	India (Mumbai)
P08	Male	35	Post Doc, Monk	Devout	India (Delhi)
P09	Female	25	Student (PG 1st Yr)	Devout	Diaspora (London)
P10	Female	45	Life Coach	Devout	Diaspora (Seattle)
P11	Female	48	Co-Founder and CEO	Spiritual-but not religious	India (Mumbai)
P12	Female	36	Director (Corporate)	Devout	India (Mumbai)
P13	Female	33	Housewife	Neutral	India (Visakhapatnam)
P14	Female	30	PhD in Psychology (Part-time)	Devout	Diaspora (Ukraine)
P15	Female	28	Student (PhD)	Devout	Diaspora (Adelaide)
P16	Male	20	Student (UG, 2nd Yr)	Neutral	India (Mumbai)
P17	Female	59	Housewife	Devout	India (Mumbai)
P18	Male	25	Software Engineer	Spiritual-but not religious	India (Mumbai)
P19	Male	32	Partner and Fund Manager	Spiritual-but not religious	India (Mumbai)
P20	Male	24	Software Engineer	Devout	India (Mumbai)
P21	Female	29	Lawyer	Spiritual-but not religious	India (Mumbai)
P22	Female	32	Student (PhD)	Devout	Diaspora (Cincinnati, USA)
P23	Female	57	Nutritionist	Devout	India (Mumbai)
P24	Female	22	Student (MBA, 1 Yr)	Devout	India (Vadodara)
P25	Male	44	Principal engineer	Devout	India (Bangalore)
P26	Female	32	Entrepreneur	Spiritual-but not religious	India (Mumbai)

## Appendix B

**Sample Interview Questions**

1. Can you share a recent emotional challenge and how you responded?
2. What role does spirituality or your core values play in your daily life?
3. How do you understand the idea of ā*tma-bodha* (self-awareness)?
4. How do you deal with criticism or emotional turbulence (e.g., *samatva*)?
5. Can you give an example of working without attachment to results (*niṣkāma-karma*)?
6. How do you apply teachings from the *Bhagavad Gitā* during stress or decision-making?
7. What does *bhakti* or devotion mean to you, if at all?
8. How has this inner orientation affected your wellbeing or relationships?

*Note: The questions were adapted depending on the participant's spiritual orientation (devout, moderate, neutral), and some prompts were adjusted in real-time for relevance and comfort*.
